# Management of medically inoperable and tyrosine kinase inhibitor-naïve early-stage lung adenocarcinoma with epidermal growth factor receptor mutations: a retrospective multi-institutional analysis

**DOI:** 10.1186/s12885-020-07122-7

**Published:** 2020-07-13

**Authors:** Yuemei Sun, Mengwan Wu, Mingxiu Zhou, Xing Luo, Yan Guo, Hansong Bai, Zican Zhang, Wei Tian, Xiaoshan Wang, Yifeng Bai, Xueqiang Zhu, Haixia Pan, Ying Deng, Honglin Hu, Jianling Xia, Xinbao Hao, Liangfu Han, Min Wei, Yingyi Liu, Ming Zeng

**Affiliations:** 1 Cancer Center, Sichuan Academy of Medical Sciences & Sichuan Provincial People’s Hospital, School of Medicine, University of Electronic Science and Technology of China, Chengdu, Sichuan China; 2 North Sichuan Medical College, Nanchong, Sichuan China; 3 School of Medicine, University of Electronic Science and Technology of China, Chengdu, Sichuan China; 4 Sino-America Cancer Center, Hainan Medical University, First Affiliated Hospital of Hainan Medical College, Haikou, Hainan China; 5 Cancer Center, BoaoEvergrande International Hospital, Qionghai, Haikou, Hainan China; 6 Cancer Center, Ziyang People’s Hospital, Ziyang, Sichuan China; 7 Dept of Radiation Oncology, Sichuan Friendship Hospital, Chengdu, Sichuan, China

**Keywords:** EGFR, Inoperable, Lung adenocarcinoma, Radiation therapy, TKI

## Abstract

**Background:**

The clinical value of combined local radiation and epidermal growth factor receptor (EGFR)-tyrosine kinase inhibitors (TKIs) for medically inoperable and TKI-naïve early-stage lung adenocarcinoma patients with EGFR mutations has not yet been determined. In this study, we aimed to pool multi-institutional data to compare the therapeutic effect of EGFR-TKI treatment alone and combined radiation and TKI treatment on the survival outcomes in this patient subgroup.

**Methods:**

A total of 132 cases of medically inoperable stage I to III EGFR mutant lung adenocarcinoma were retrospectively reviewed based on data from 5 centers. Among these patients, 65 received combined radiation and EGFR-TKI therapy (R + TKI) (49.2%), while 67 received EGFR-TKI (50.8%) treatment alone. All patients were followed until death.

**Results:**

For the R + TKI group, the median overall survival (OS) after primary therapy was 42.6 months, while that of the TKI alone group was 29.4 months (log-rank *p < 0.*001). In terms of progression-free survival (PFS), the median PFS in these two treatment groups was 24 months and 14.7 months respectively (log-rank *p < 0.*001). Multivariate analysis showed that R + TKI was independently associated with improved OS (adjusted HR 0.420; 95% CI 0.287 to 0.614; *p < 0.*001) and PFS (adjusted HR 0.420; 95% CI 0.291 to 0.605; *p < 0.*001) compared to TKI alone. Subgroup analysis confirmed the significant OS benefits in stage III patients and RFS benefits in stage II/III patients.

**Conclusions:**

Upfront radiation to primary sites with subsequent TKI treatment is a feasible option for patients with medically inoperable EGFR-mutant non-small-cell lung carcinoma (NSCLC) during first-line EGFR-TKI treatment, with significantly improved PFS and OS compared with those yielded by TKI treatment alone.

## Background

Lung cancer remains the most commonly diagnosed cancer and the leading cause of cancer-related death worldwide. GLOBOCAN (2018) estimates that lung cancer accounts for approximately 18.4% of the total cancer deaths [1]. NSCLC is the dominant type of lung cancer, in which 40% of patients need surgical resection for localized disease. However, certain patients are medically inoperable or unwilling to receive dramatically invasive procedures.

Lung adenocarcinoma is one of the most common subtypes of NSCLC. In recent decades, it was found that 10–15% of Caucasian patients harbor epidermal growth factor receptor (EGFR) mutations [2, 3]. In comparison, this rate can be as a high as 60% in patients from Eastern Asia [4]. This group of patients has a higher likelihood of being treated with EGFR targeted therapies (typically EGFR tyrosine kinase inhibitors (TKIs) because of the high tolerance, overall response rate (ORR) and prolonged progression -free Survival (PFS) [5].In addition, in patients with brain metastasis, the use of TKIs might potentiate the effect of radiation therapy [6, 7].

Historically, medically inoperable lung cancer patients have been treated with primary radiation therapy, stereotactic body radiotherapy (SBRT) for stage I/II and concurrent external beam radiotherapy (EBRT) with chemotherapy for stage III [8]. The clinical value of the adjuvant use of TKIs in these patients has been gradually revealed. Two previous retrospective studies explored the effect of TKI treatment on survival outcomes in patients with resected lung adenocarcinoma and EGFR mutations from the US [9, 10]. Their findings suggested that in resected stage I-III lung adenocarcinoma, adjuvant TKI might significantly improve the disease-free survival (DFS) rate compared to patients who do not receive adjuvant TKI [9, 10]. This trend was confirmed by another recent retrospective study based on a Chinese patient database, which had a higher prevalence of EGFR mutation [11]. More recently, one phase III trial evaluated the adjuvant use of gefitinib in patients with completely resected stage II-IIIA (N1-N2) EGFR-mutant NSCLC [12]. Their data confirmed that compared to the adjuvant chemotherapy grounp, the adjuvant gefitinib group had superior DFS, reduced toxicity, and improved quality of life compared to the adjuvant chemotherapy group [12]. These findings imply that EGFR-targeting therapy might have clinical value for treating early-stage EGFR- mutatnt patients. However, the necessity of local radiation for this subgroup of patients is not certain. Therefore, there has been enormous interest in testing the efficacy of local radiation in addition to EGFR-TKIs. Although the radiation with TKI have been published [13], there are no randomized data available to study EGFR-TKI versus combined radiation and TKI.

In this study, we aimed to pool multi-institutional data and to compare the influence of EGFR-TKI alone with that of combined radiation and TKI on the survival outcomes in TKI-naïve early-stage lung adenocarcinoma patients with EGFR mutations.

## Methods

### Inclusion and exclusion criteria

After approval by the Sichuan Academy of Medical Sciences and Sichuan Provincial People’s Hospital Investigation Committee, patient information was gathered from five academic centers. Patients who had medically inoperable stage I to III EGFR mutant lung adenocarcinoma between January 1, 2010, and December 31, 2011, were identified. Diagnosis and staging of primary tumors were performed according to AJCC version 8. The inclusion criteria were TKI-naïve patients with newly diagnosed stage I to III disease who refused either surgery or chemotherapy for clinical node-positive disease, or patients who could not tolerate surgery but had resectable N disease. Patients who were treated with radiation followed by EGFR-TKI or with EGFR-TKI followed by radiation at primary site progression (named R + TKI) and patients who received only EGFR-TKI therapy (named as TKI alone) were included. The exclusion criteria were as follows: patients who had prior EGFR-TKI use patient who had EGFR-TKI resistance mutations patients for whom EGFR-TKI treatment was not performed after radiotherapy patients who received chemotherapy or immunotherapy, or received third-generation TKIs such as osimertinib for T790M mutation during TKI treatment patients with brain, visceral or bony metastases or patients who were missing covariable data or had an insufficient follow up time. To lessen a potentially confounding variable, patients who received surgical resection or neoadjuvant chemo- or immunetherapy at the time of initial treatment were also excluded. Radiation included stereotactic body radiation therapy (SBRT) or conventional external beam radiation therapy (EBRT). The SBRT dose ranged from 10 to 18 Gy in 3 to 5 fractions, while conventional EBRT ranged from 50 to 74 Gy in 25 to 35 fractions. The site of radiotherapy was the primary lesion. Tumor response was assessed using RECIST1.1, an evaluation criterion for the efficacy of solid tumors. Follow-up after treatment ocuurred once every 4 months in the first year, once every 6 months in the second and third years, and once every year in the fourth and fifth years.

### Data extraction

The following variables were collected for subsequent analysis: age, gender, clinical stage, smoking history, EGFR mutation, clinical stages, type of RT delivered, name of the EGFR-TKI, and type of systemic therapy after progressing on EGFR-TKI treatment. Systemic disease status was assessed by the presence or absence of brain, or visceral or bone metastases at the time of initial treatment. The site of first progression after primary site radiation (SBRT or conventional) was identified. The date of initial cancer diagnosis; clinical stage; RT treatments; systemic therapy treatments; distant metastases including intracranial; visceral or bony disease; most recent follow-up; and death were recorded.

Positron emission tomography-computed tomography (CT) and CT scans of the chest, abdomen, pelvis, and bone scan were reviewed to ascertain the clinical stage, and any uncertain lesions required biopsies to rule out metastases. Pulmonary function tests (PFT) were performed before and after chest radiation to monitor the changes in lung function for all SBRT patients. Mediastinal node disease was evaluated by combinating PET and contract CT, and suspicious nodes were biopsied by endobronchial ultrasound (EBUS). EGFR mutations were evaluated by polymerase chain reaction amplification through next-generation sequencing (NGS) techniques. Exons 18 to 21 were analyzed for the following mutation; a deletion on exon 19 (E746-A750), or a point mutation on exon 21 (L858R). The stduy excluded ALK rearrangements, Rose1 mutations and rare mutations. Tumor response was assessed using RECIST1.1, an evaluation criterion for the efficacy of solid tumors. Follow-up after treatment was once every 4 months in the first year, once every 6 months in the second and third years, and once every year in the fourth and fifth years.

### Statistical analysis

Statistical analyses were conducted using SPSS 25.0 software (SPSS, Chicago, IL, USA) and GraphPad Prism 7.04 (GraphPad Inc., La Jolla, CA). Characteristics of patients (categorical variables) in the two groups were analyzed by the χ^2^ test with two-sided Fisher’s exact test. Kaplan-Meier OS curves and PFS curves were generated. Log-rank testing was used to assess the differences between the curves. OS was defined from the date of initial diagnosis until the date of death. PFS was defined from the date of initial diagnosis until the date of recurrence of a prior irradiated primary site(s) or the development of a new lesion. Using Cox proportional hazards analysis, univariable and multivariable variables were examined for the factors associated with OS and PFS. A value of *p < 0.*05 was considered statistically significant.

## Results

After applying the inclusion and exclusion criteria mentioned above, 132 patients from five centers were included in this study. Among the patients, 65 patients received combined radiation and EGFR-TKI therapy (49.2%), while 67 patients received EGFR-TKI (50.8%) treatment alone. All patients were followed until death. Patient characteristics are given and compared in Table 1. The age (mean ± SEM) before therapy for the R + TKI group and TKI alone group was 70.2 ± 1.12 and 70.88 ± 1.01 years respectively. The R + TKI group included 13 stage I, 16 stage II and 36 stage III patients, while the TKI alone group included 8 stage I, 12 stage II and 47 stage III patients (Table 1). The χ^2^ test did not reveal any significant differences between the parameters, including age, gender, stage, nodal status, EGFR mutations and type of radiation therapy (*p* > 0.05) (Table 1).

**Table 1:** Comparison of the clinicopathological parameters between the R + TKI and TKI alone groups

Parameters	Treatment		***P*** value
	R + TKI (***N*** = 65)	TKI alone (***N*** = 67)	
**Age (mean ± SEM)**	70.2 ± 1.115	70.88 ± 1.008	0.65
**Gender**			
Female	28	27	0.73
Male	36	40	
No data	1	0	
**Pathological stages**			
I	13	8	0.20
II	16	12	
III	36	47	
**Nodal status**			
N0	18	18	1.00
N1/N2	47	49	
**EGFR mutations**			
exon 19	54	58	0.86
exon 20	6	5	
exon 21	6	5	
**Radiation therapy**			
EBRT	61	0	0.99
SBRT	4	0	

*EBRT* External beam radiation therapy, *SBRT* Stereotactic body radiation therapy

### Comparison of the survival outcomes between the two therapeutic strategies

**Fig. 1:**
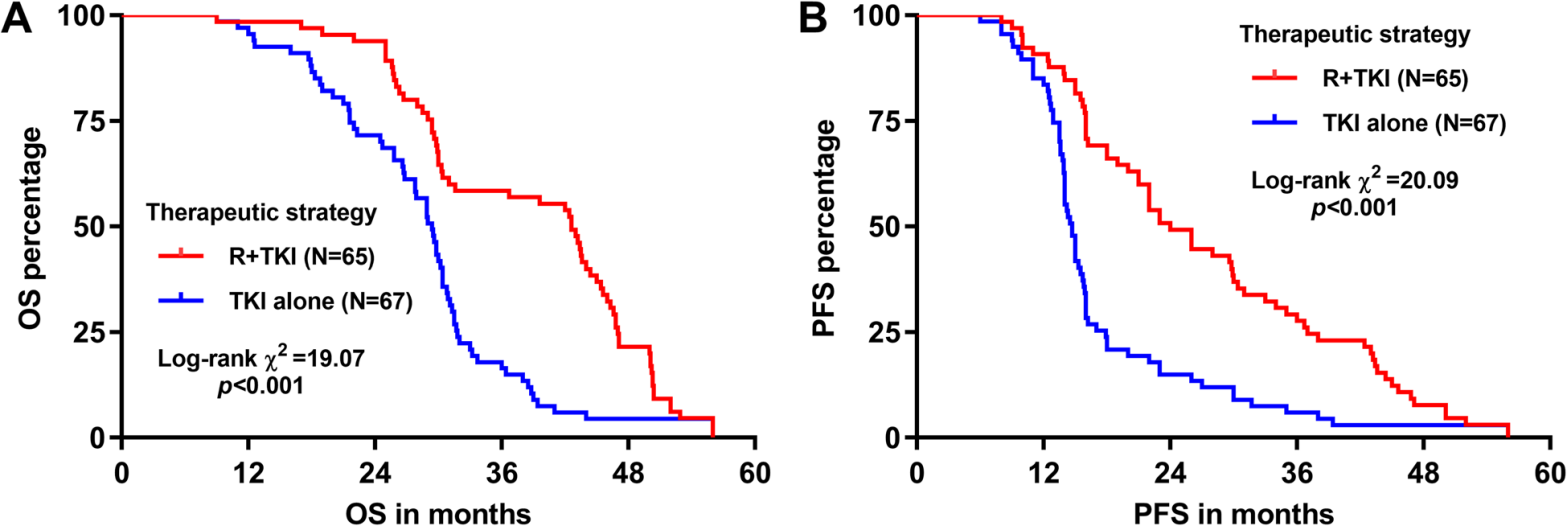
Comparison of survival outcomes in patients who received R + TKI or TKI alone. Kaplan-Meier OS (**a**) and PFS (**b**) curves were generated. Patients included were separated into R + TKI (*N* = 65) and TKI-alone (*N* = 67) groups

For the R + TKI group, the median OS after primary therapy was 42.6 months, while that of the TKI alone group was 29.4 months (log-rank *p < 0.*001; Fig. 1a). The median PFS in these two treatment groups was 24 months and 14.7 months respectively (log-rank *p < 0.*001; Fig. 1b). In the univariate analysis, advanced stages, EBRT and TKI alone were associated with significantly shorter OS. Following the multivariate analysis R + TKI was independently associated with improved OS relative to TKI alone (adjusted HR 0.420; 95% CI 0.287 to 0.614; *p < 0.*001; Table 2), after controlling for other significant covariables. In addition, multivariate analysis also showed that R + TKI was independently associated with improved PFS, compared to TKI alone (adjusted HR 0.420; 95% CI 0.291 to 0.605; *p < 0.*001; Table 3), after controlling for the significant covariables. Controlled covariables included age gender, nodal status, stages, RT strategy.

**Table 2:** Univariate and multivariate analysis of OS

Characteristics	Univariate analyses				Multivariate analyses			
	***P***	HR	95% CI lower	95% CI upper	***P***	HR	95% CI lower	95% CI upper
**Age (Continuous)**	0.688	0.996	0.977	1.015				
**Gender**								
Male		1.000						
Female	0.872	0.972	0.686	1.376				
**Nodal status**								
N0		1.000						
N1/N2	0.074	1.426	0.966	2.107				
**Stages**								
I		1.000						
II	0.110	1.592	0.900	2.818				
III	**< 0.001**	2.756	1.655	4.588	**0.002**	2.314	1.368	3.914
**RT strategy**								
SBRT		1.000						
EBRT	**0.015**	2.344	1.184	4.642	**0.023**	2.289	1.120	4.676
**Therapeutic strategy**								
TKI alone		1.000						
R + TKI	**< 0.001**	0.466	0.325	0.669	**< 0.001**	0.420	0.287	0.614

*HR* Hazard ratio, *CI* Confidence interval, *SBRT* Stereotactic body radiation therapy, *EBRT* External beam radiation therapy, *R + TKI* Combined radiation and TKI

**Table 3:** Univariate and multivariate analysis of PFS

Characteristics	Univariate Analyses				Multivariate Analyses			
	***P***	HR	95% CI lower	95% CI upper	***P***	HR	95% CI lower	95% CI upper
**Age (Continuous)**	0.355	0.991	0.973	1.010				
**Gender**								
Male		1.000						
Female	0.743	1.060	0.748	1.502				
**Nodal status**								
N0		1.000						
N1/N2	**0.007**	1.800	1.171	2.767	0.283	0.741	0.428	1.282
**Stages**								
I		1.000						
II	**0.004**	2.604	1.347	5.033	**0.018**	2.781	1.191	6.492
III	**< 0.001**	3.408	1.918	6.053	**0.002**	3.474	1.599	7.548
**RT strategy**								
SBRT		1.000						
EBRT	**0.001**	3.160	1.572	6.355	**0.005**	2.779	1.353	5.710
**Therapeutic strategy**								
TKI alone		1.000						
R + TKI	**< 0.001**	0.465	0.326	0.662	**< 0.001**	0.420	0.291	0.605

### Subgroup analyses

**Fig. 2:**
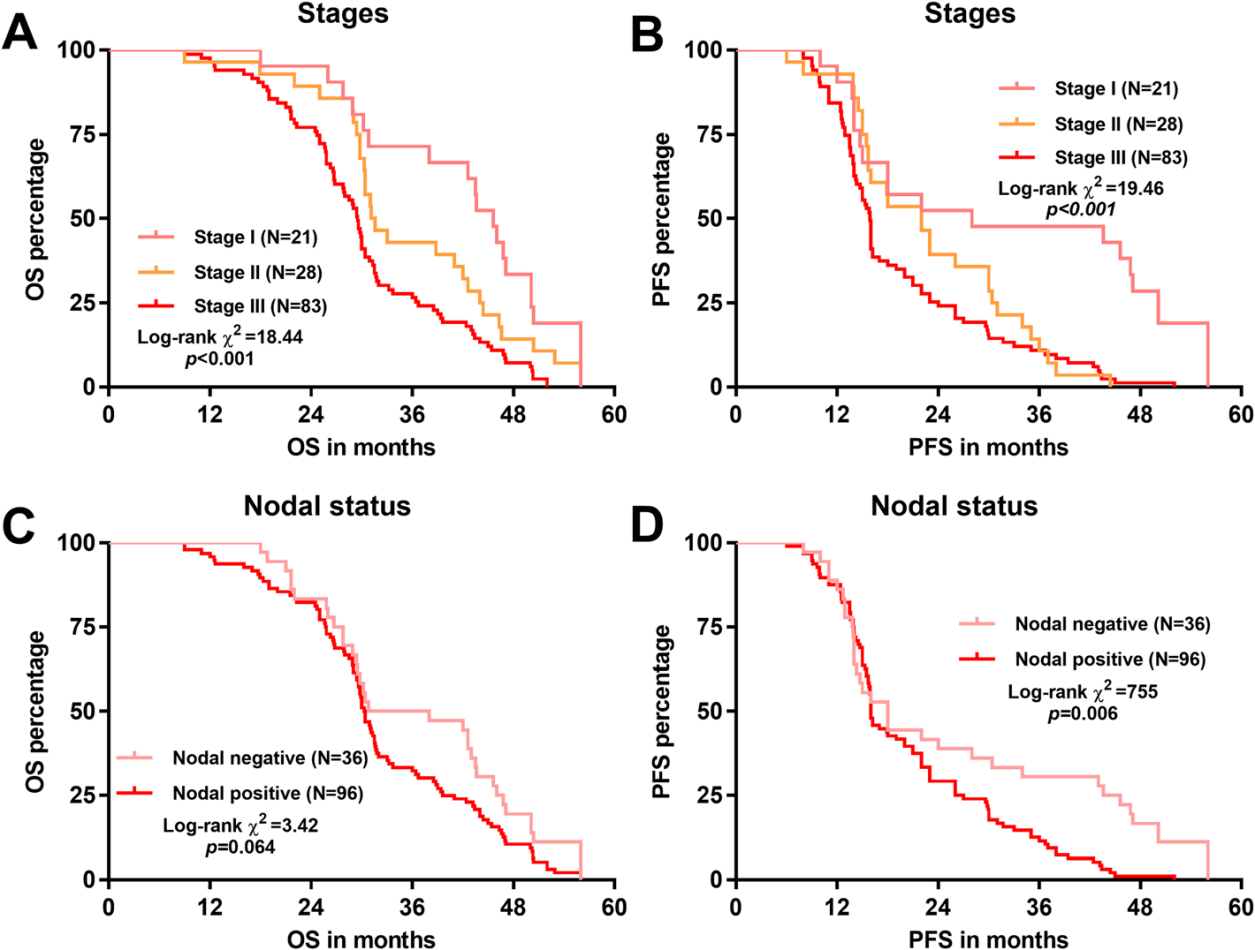
Comparison of survival outcomes in patients with different pathological stages and nodal status. Kaplan-Meier OS (**a** and **c**) and PFS (**b** and **d**) curves were generated. Patients were grouped according to their pathological stage (**a-b**) or nodal status (**c-d**)

To explore the potential variations of the survival benefits in patients with different clinicopathological parameters, we subdivided patients according to their pathological stages, T stages and nodal status. Regardless of the therapeutic strategy, patients with higher pathological stages had a significantly shorter OS and PFS (log-rank *p < 0.*001; Fig. 2a-b). In comparison, nodal positive cases had inferior OS at the margin level of significance (log-rank *p = 0.*064, Fig. 2c) and significantly shorter PFS (log-rank *p = 0.*006, Fig. 2d).

**Fig. 3:**
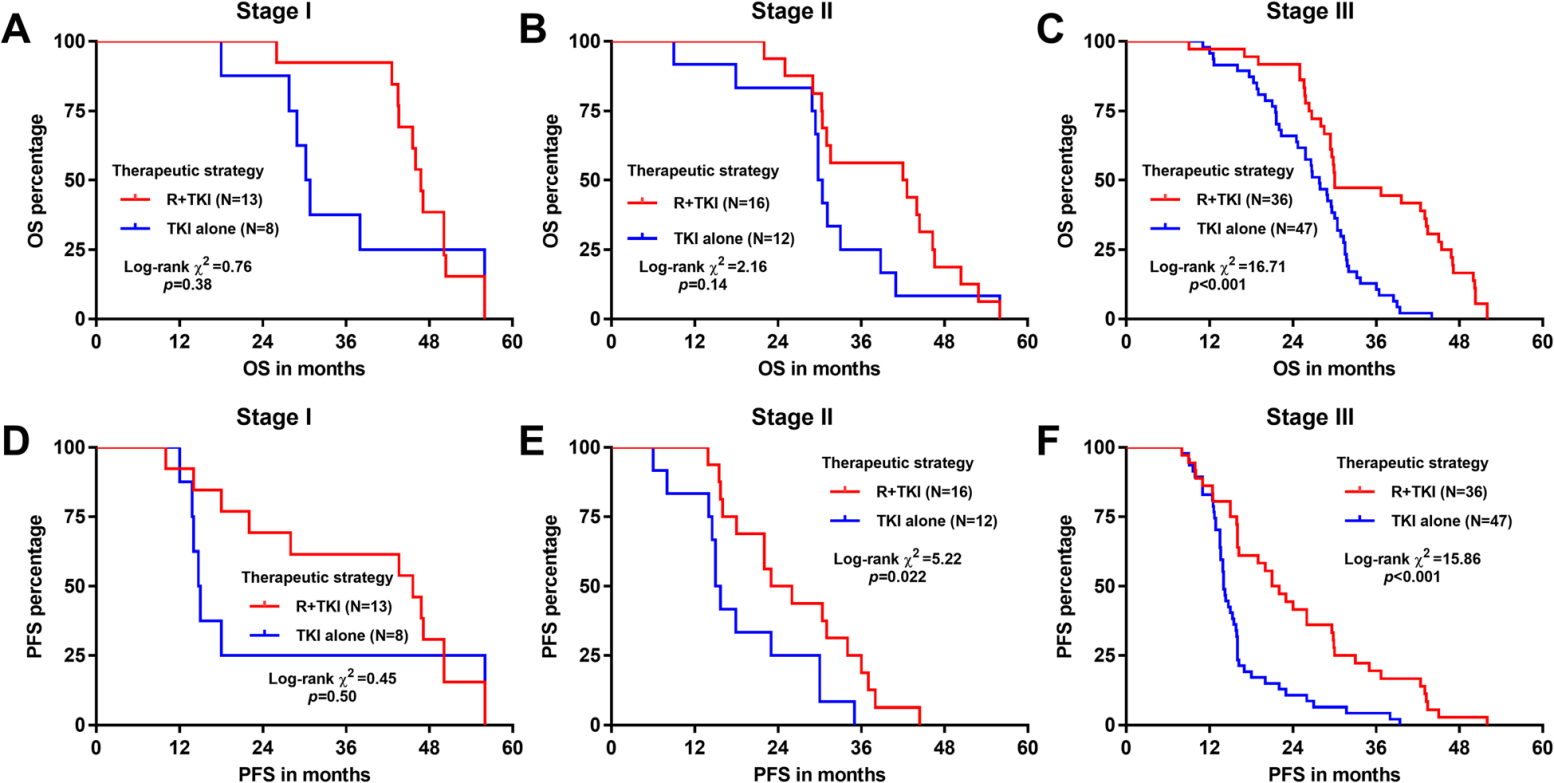
Comparison of OS and RFS in patients in different pathological stages. Kaplan-Meier OS (**a-c**) and PFS (**d-f**) curves were generated. Patients were grouped according to their pathological stages. Kaplan-Meier PFS curves were generated

Those stage I patients who had the best survival outcomes did not have improved OS or PFS when they received combined radiation and TKI therapy (log-rank *p = 0.*38 and 0.50 respectively, Fig. 3a and c). In stage II patients, although R + TKI did not improve OS (log-rank *p = 0.*14, Fig. 3b), it substantially prolonged PFS (log-rank *p = 0.*022, Fig. 3e). In stage III patients who had the worst prognosis, R + TKI significantly improved both OS and PFS, compared to TKI alone (log-rank *p < 0.*001, Fig. 3c and f).

The median OS of the stage III R + TKI group was 30 months, which was similar to that of stage I and II patients who received TKI alone (30.5 months and 30.1 months respectively). In contrast, the median OS of stage III TKI alone was 27.8 months. The median PFS of the stage III R + TKI group was 21.5 months, which was longer than that of stage I and II patients who had TKI alone (14.85 months and 15.35 months respectively). In comparison, the median PFS of stage III TKI alone was only 14 months.

**Fig. 4:**
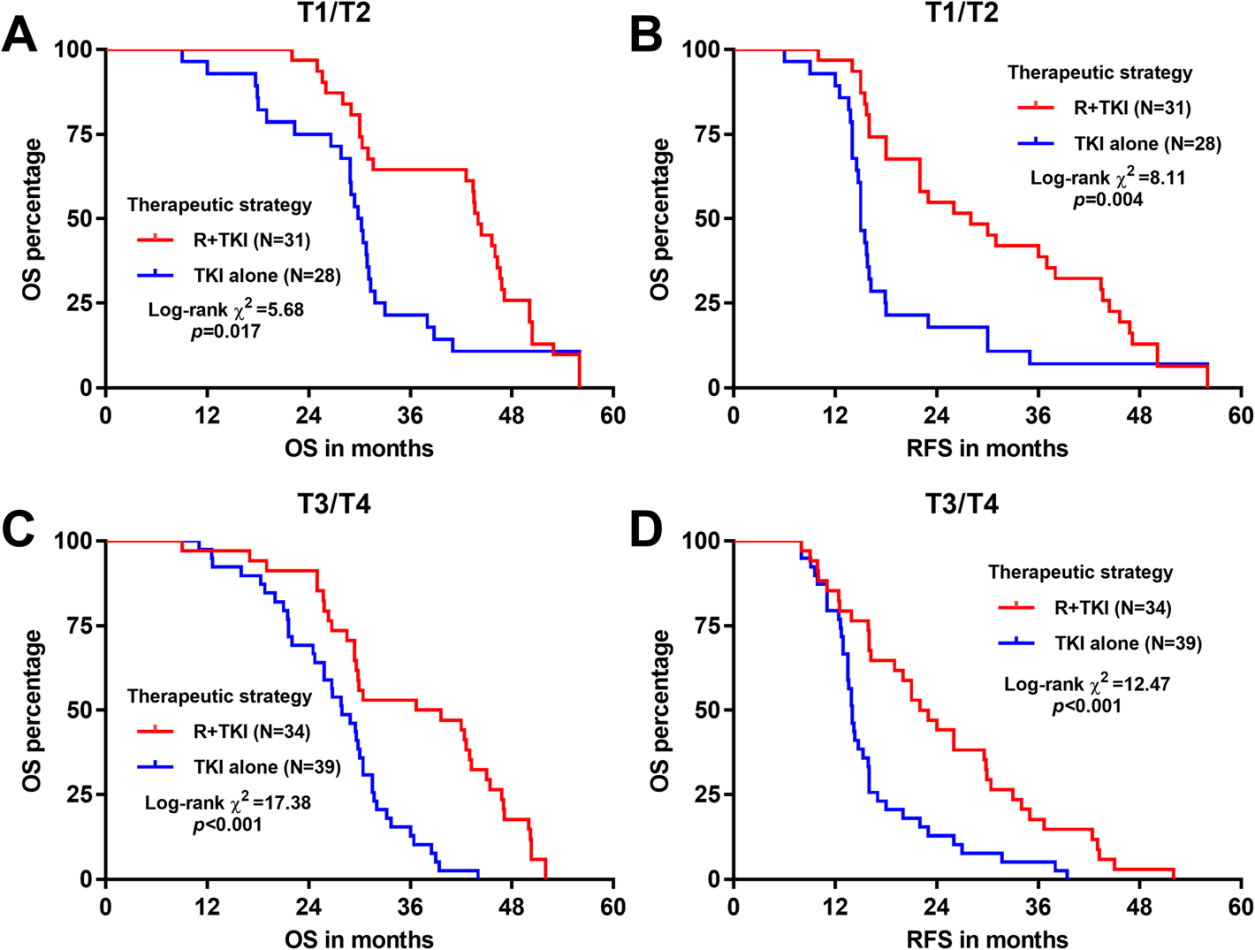
Comparison of PFS in patients in early and late T stages. Kaplan-Meier OS (**a** and **c**) and PFS (**b** and **d**) curves were generated. Patients were separated into early T stages (T1/T2) (**a-b**) and late T stages (T3/T4) (**c-d**) groups

**Fig. 5:**
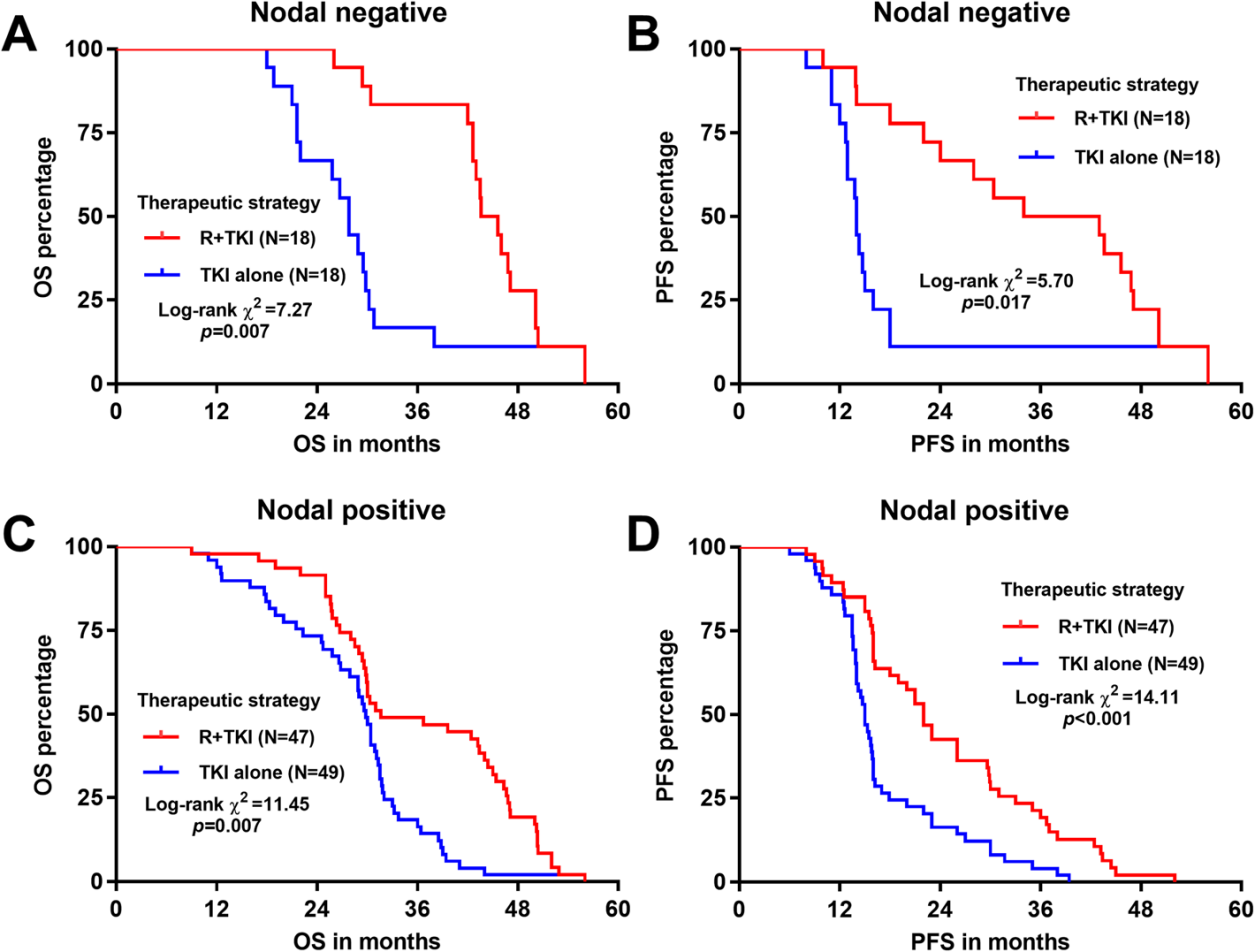
Comparison of OS and PFS in patients with different nodal statuses. Kaplan-Meier OS (**a** and **c**) and PFS (**b** and **d**) curves were generated. Patients were separated into nodal negative (**a-b**) and nodal positive (**c-d**) groups

When dividing the patients according to their T stages, R + TKI significantly improved both OS and RFS in the early T stage (T1/T2) (log-rank *p = 0.*017 and 0.004 respectively, Fig. 4a-b) and the late T stage (T3/T4) (log-rank *p* < 0.001, Fig. 4c-d) cases. In the subgroups divided by nodal status, R + TKI also significantly improved OS and PFS in nodal negative cases (log-rank *p = 0.*007 and 0.017 respectively, Fig. 5a-b) and nodal positive cases (log-rank *p = 0.*007 and < 0.001 respectively, Fig. 5c-d) cases.

## Discussion

To our knowledge, this is the first study in the literature to investigate the role of radiation before starting systemic therapy with the 1st generation of TKIs in patients with NSCLC harboring with EGFR-activating mutations. For this cohort of patients, we demonstrated that TKI alone is not as effective as upfront radiation therapy followed by TKI treatment in both PFS and OS in certain pathological stages. In stage III patients, upfront RT followed by TKI significantly prolonged OS compared with the TKI alone group. Upfront radiation is also associated with improved PFS in stage II/III patients, with fewer benefits in stage II than in stage III. Moreover, the pathological parameters such as stages, performance status, age, gender, node metastases, and EGFR mutation exon location, were similar between the R + TKI and TKI alone groups, suggesting that these two groups are comparable. By performing multivariate analysis, we confirmed the prognostic significance of upfront radiation therapy followed by TKI treatment in both OS and PFS. These findings suggest that the improved OS and PFS in the upfront RT group is not secondary to the pathological parameters between patient cohorts but is due to local therapeutic treatment at primary sites.

In randomized trials, few data have compared the effect of TKIs with or without radiotherapy for stage I to III subgroup disease. Our data supported the assumption that stage I to III subgroup disease, local radiation therapy can improve survival by controlling disease progression. In the stage I/II subgroup, SBRT provides much better local control, and the benefit from TKIs is less evident than that in more progressive stage III disease. This could be a result of the high potential of radiation alone to cure early-stages disease compared with late-stage disease. Therefore, the trend toward increased OS by adding TKI to radiation is applicable to the fact that local therapy itself has less local controlling potential.

Large trials using standard first-line TKI treatment for the broad population of patients with metastatic NSCLC harboring EGFR mutations yielded a PFS between 8 and 14 months without improving OS [5, 14–18]. However, with the addition of local radiation to first-line TKIs for patients with EGFR-mutated metastatic NSCLC, both PFS and OS can be significantly improved. Gomez et al. conducted a randomized trial that compared local radiation versus maintenance treatment or observation for 49 patients with stage IV NSCLC with three or fewer metastases remaining after first-line systemic therapy [19]. Their data showed that the median PFS was significantly improved with the use of consolidation therapy (11.9 versus 3.9 months, HR = 0.35,95% CI:0.18–0.66, *p* = 0.0054). Another randomized, phase II, open-label, multicenter study (SABR-COMET) demonstrated that aggressive local radiation doubled the DFS and also dramatically improved the OS. Patients who received radiation/surgery experienced a median OS of 41.2 months vs 17.0 months among patients who received standard maintenance therapy/observation (*p* = 0.017) [20].

In this study, we confirmed the role of upfront radiation in adding a survival benefit in medical inoperable stage I to III harboring EGFR mutant NSCLC patients compared with TKI alone. In addition, the survival benefits were more evident in the late T stage or N stage. Our study is unique in a number of ways when compared with similar, recently published research: (1) the radiation as local therapy depended the stage; (2) all patients had inoperable conditions; (3) no patient received the 2nd- or 3rd -generation TKIs, which are often used in daily practice to control the drug resistance from 1st-generation TKIs after a year or so; (4) because of medical intolerance, no patients had received chemotherapy. The combination of these features made this study cohort a unique subpopulation in the lung adenocarcinoma.

This study also has several limitations. First, this was a retrospective study, with different providers of the 1st-generation of TKIs were used; second, only a small proportion of patients received SBRT. Therefore, a prospective study is needed. Currently, studies investigating both consolidative RT after TKI (NCT03256981) and concurrent radiotherapy with TKI (NCT02893332) are ongoing. Nonetheless, pending prospective validation, our results suggest that compared with TKI treatment alone, RT does significantly improve both PFS and OS in medically inoperable EGFR-mutant adenocarcinoma of the lung compared with TKI alone. Although immunotherapy is accepted as a first-line therapy, a large percentage of patients harboring EGFR NSCLC who will receive TKIs as part of their treatment. Therefore, the findings of this study will continue to be very relevant to patients with EGFR mutant NSCLC.

## Conclusions

In conclusion, upfront radiation to primary sites with subsequent TKI treatment is a feasible option for patients with mediclly inoperable EGFR-mutant NSCLC during first-line EGFR-TKI treatment, with significantly improved PFS and OS compared with those yielded by TKI treatment alone.

## Data Availability

The datasets and analyzed during the current study are available from the corresponding author on reasonable request.

## Abbreviations

CT: Computed tomography
EGFR: Epidermal growth factor receptor
EBRT: External beam radiotherapy
EBUS: Endobronchial ultrasound
NSCLC: Non-small-cell lung carcinoma
NGS: Next-generation sequencing
OS: Overall survival
ORR: Overall response rate
PFS: Progression-free survival
PFT: Pulmonary function tests
SBRT: Stereotactic body radiotherapy
SABR: Stereotactic ablative radiation therapy
TKI: Tyrosine kinase inhibitor
